# Demographics and Clinical Features of Postresuscitation Comorbidities in Long-Term Survivors of Out-of-Hospital Cardiac Arrest: A National Follow-Up Study

**DOI:** 10.1155/2017/9259182

**Published:** 2017-02-13

**Authors:** Chih-Pei Su, Jr-Hau Wu, Mei-Chueh Yang, Ching-Hui Liao, Hsiu-Ying Hsu, Chin-Fu Chang, Shou-Jen Lan, Chiao-Lee Chu, Yan-Ren Lin

**Affiliations:** ^1^Department of Health Care Administration, Asia University, Taichung, Taiwan; ^2^Department of Emergency Medicine, Changhua Christian Hospital, Changhua, Taiwan; ^3^Department of Nursing, Changhua Christian Hospital, Changhua, Taiwan; ^4^Department of Long Term Care, National Quemoy University, Kinmen, Taiwan; ^5^School of Medicine, Kaohsiung Medical University, Kaohsiung, Taiwan; ^6^School of Medicine, Chung Shan Medical University, Taichung, Taiwan

## Abstract

The outcome of patients suffering from out-of-hospital cardiac arrest (OHCA) is very poor, and postresuscitation comorbidities increase long-term mortality. This study aims to analyze new-onset postresuscitation comorbidities in patients who survived from OHCA for over one year. The Taiwan National Health Insurance (NHI) Database was used in this study. Study and comparison groups were created to analyze the risk of suffering from new-onset postresuscitation comorbidities from 2011 to 2012 (until December 31, 2013). The study group included 1,346 long-term OHCA survivors; the comparison group consisted of 4,038 matched non-OHCA patients. Demographics, patient characteristics, and risk of suffering comorbidities (using Cox proportional hazards models) were analyzed. We found that urinary tract infections (*n* = 225, 16.72%), pneumonia (*n* = 206, 15.30%), septicemia (*n* = 184, 13.67%), heart failure (*n* = 111, 8.25%) gastrointestinal hemorrhage (*n* = 108, 8.02%), epilepsy or recurrent seizures (*n* = 98, 7.28%), and chronic kidney disease (*n* = 62, 4.61%) were the most common comorbidities. Furthermore, OHCA survivors were at much higher risk (than comparison patients) of experiencing epilepsy or recurrent seizures (HR = 20.83; 95% CI: 12.24–35.43), septicemia (HR = 8.98; 95% CI: 6.84–11.79), pneumonia (HR = 5.82; 95% CI: 4.66–7.26), and heart failure (HR = 4.88; 95% CI: 3.65–6.53). Most importantly, most comorbidities occurred within the first half year after OHCA.

## 1. Introduction

The outcome of patients who suffer from out-of-hospital cardiac arrest (OHCA) is very poor, and postresuscitation comorbidities increase the long-term mortality [1–4]. The incidences of OHCA clearly differ worldwide (5.7, 38, and 32.7 per 100,000 population each month in the United States, Europe, and Korea, resp.) [5]; however, the average rate of survival to discharge is mostly less than 10% (10.6% in the United States, 5% in Japan, and only 0.5% in Malaysia) [6, 7]. Furthermore, some previous studies reported a one-year survival rate of OHCA of only 7.7% to 11.5% on average [8, 9]. Among these discharged patients, up to 16% of them did not survive over one year [4, 10, 11]. One major reason for the large decrease in rate between survival to discharge and one-year survival is the development of postresuscitation comorbidities [4, 12]. Comorbidities can not only be associated with postcardiac arrest syndrome (systemic ischemia/reperfusion injuries, hypoxia, acidosis, and immune/inflammatory over reactions) but can also be related to poor support during the postresuscitation period [8, 9, 13].

Some economic studies have noted that the medical resources consumed for resuscitating OHCA patients and providing long-term care are quite high [14, 15]. One previous study in Taiwan reported that an OHCA survivor would cost approximately USD$ 14,000 on average for in-hospital resuscitation and postresuscitation comorbidities after discharge [16]. In the United Kingdom, the in-hospital cost of each OHCA patient is generally as high as USD$ 61,000 [17]. In fact, for each OHCA survivor, it would cost approximately $USD 6600 to 10,000 per life-year saved for postresuscitation care [18, 19].

Since postresuscitation comorbidities require high medical resources and decrease the chance of long-term survival, knowledge regarding the prevention or early treatment of comorbidities should be emphasized. However, most current studies concerning OHCA have focused on increasing survival rates or neurological outcomes [20, 21]. Information regarding the prevalence of postresuscitation comorbidities, demographics, and patient characteristics is not well known; in particular, data on long-term follow-ups are lacking. In this study, we aim to analyze new-onset postresuscitation comorbidities in patients who survived over one year after OHCA using the Taiwan national health care system database.

## 2. Methods and Materials

### 2.1. Data Source

The Taiwan National Health Insurance (NHI) database was used in this study. The NHI has had nearly 100% coverage of the population since its initiation in 1995 [22]. Since 2000, the NHI has collected all declaration data at the end of every year and derived a corresponding database. The NHI database was created to develop public health policy and clinical research. We obtained our data from the Health and Welfare Data Science Center (HWDC) of Taiwan, a database from the Ministry of health and welfare developed by the NHI program, to study postresuscitation comorbidities in OHCA survivors through a secondary analysis (all data were deidentified) of the study period (January 1, 2011, to December 31, 2012). The registered populations in this database were 22,263,417 and 22,362,328 on December 31, 2011, and December 31, 2012, respectively. All OHCA survivors in this period were followed for postresuscitation comorbidities until December 31, 2013 (the same end point).

### 2.2. Ethical Approval

Data in the NHI database that could be used to identify patients or care providers were scrambled before being sent to the database and were further concealed before being released to the researchers. Therefore, it was impossible to identify individuals from this database by querying the data alone. This study protocol was approved by the Institutional Review Board (IRB) of Changhua Christian Hospital (permission code: 150117).

### 2.3. Sample Selection and Definition

**Figure 1:**
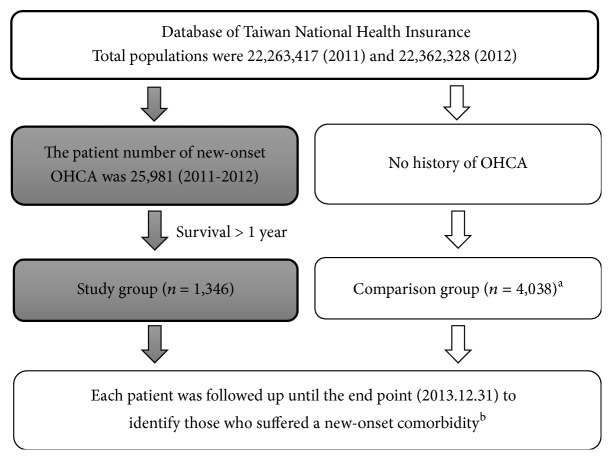
The selection methods of study and comparison patients. ^a^Comparison patients were adjusted by age, gender, and follow-up periods to patients in the study group. They three-times outnumbered the study group. ^b^New-onset comorbidities in the study group were defined as diseases developed after one month of survival.

This is a retrospective cohort study. Two groups (study and comparison groups) were classified in this study. The two groups were both extracted from the database during the study period (January 1, 2011, to December 31, 2012). Each patient had been followed for new-onset comorbidities (or diseases in the comparison group) until the end point (December 31, 2013). The selection methods of the study and comparison groups are summarized in Figure 1.

#### 2.3.1. Definition of Inclusion Criteria of Study Group

Patients in the study group were extracted from the database during the study period using the following two major criteria.

1. Patients who suffered from their first experience of OHCA and visited the emergency department (ED). The OHCA event was screened by using the International Classification of Diseases, 9th revision, clinical modification codes (ICD-9-CM 798, 798.1, 798.2, 798.9, 799, 799.0, 799.1, and 427.5) in the ED.
2. Patients who survived over one year after suffering from an OHCA event.

#### 2.3.2. Definition and Inclusion Criteria of the Comparison Group

Comparison patients were randomly selected from the remaining NHI beneficiaries registered in the database. Patients without OHCA were selected as the comparison group after adjusting their age, gender, and follow-up periods to patients in the study group. They three-times outnumbered the study group [23, 24].

#### 2.3.3. Exclusion Criteria

Patients with the following conditions were excluded from this study both in the study and control groups.

1. Patients who had any history of a cardiac arrest event before the study period.
2. Patients who had incomplete medical records.

#### 2.3.4. Definition of Postresuscitation Comorbidities

In the study group, postresuscitation comorbidities were defined as a new-onset disease developing one month after survival from OHCA (diseases developed in the first month after OHCA might be directly associated with the original etiology of OHCA; therefore, these diseases were not included). In the comparison group, comorbidities were new-onset diseases developing during the follow-up period.

### 2.4. Statistical Analysis

The study and comparison groups were selected with SAS (SAS Institute Inc., Cary, NC, USA). The SAS programming language and the results of the analysis were routinely checked by supervisors of the database to ensure that all information was deidentified. Demographics, patient characteristics, and personal histories were reported as the number, percentage, or mean ± standard deviation (SD). Chi-squared test and *t*-tests were used to analyze the demographics and postresuscitation comorbidities between the study (OHCA survivors) and comparison groups (without OHCA). Furthermore, Chi-squared test was used to analyze the characteristics of the three most common postresuscitation comorbidities in the two groups. Detailed descriptions regarding classifications of the demographic data have been addressed in previous studies (including classifications of patient's economic level, degree of their city urbanization, and location of their residence in terms of geographic regions in Taiwan) [25, 26]. To analyze the risk of suffering from these comorbidities, crude hazard ratios (HRs) were also calculated (Cox proportional hazards models). Finally, time-related factors associated with the occurrence of postresuscitation comorbidities at different time points in OHCA survivors and comparison patients were also reported. *p* values < 0.05 were considered statistically significant.

## 3. Results

### 3.1. Demographics and Personal Characteristics

The total number of OHCA patients was 13,365 and 12,619 in 2011 and 2012, respectively. The mean incidence of OHCA was 4.8 per 100,000 population (per month). The mean one-year survival rate was only 5.2%. In our study, 1,346 long-term OHCA survivors were enrolled in the study group, whereas 4,038 patients were in the comparison groups. Their characteristics, personal histories, and economic levels are shown in Table 1. Personal history differed significantly between the two groups. OHCA survivors more frequently had histories of cardiac disease, diabetes, hypertension, renal failure, liver cirrhosis, and stroke (all *p* < 0.001). Moreover, OHCA survivors had a clearly lower economic status than comparison patients.

### 3.2. Postresuscitation Comorbidities

Among the OHCA survivors, the most common postresuscitation comorbidities were urinary tract infections, followed by pneumonia, septicemia, essential hypertension, heart failure, gastrointestinal hemorrhage, epilepsy or recurrent seizures, and chronic kidney disease. Except for essential hypertension (*p* = 0.38), OHCA survivors had significantly higher occurrences than comparison patients of all comorbidities (all *p* < 0.001) (Table 2).

### 3.3. The Three Most Common Comorbidities between OHCA Survivors and Comparison Patients

The three most common comorbidities were all infection-related diseases (urinary tract infections, pneumonia, and septicemia). Detailed data are shown in Table 3. Of the comparing patients, these three comorbidities were more predominant in the older age group (<75 years) (all *p* < 0.001). However, most OHCA survivors with the three comorbidities were younger than 60 years.

### 3.4. The Risk of Suffering from Postresuscitation Comorbidities

The HRs of postresuscitation comorbidities during the follow-up period were significantly higher for urinary tract infections (HR = 2.83; 95% CI: 2.37–3.37), pneumonia (HR = 5.82; 95% CI: 4.66–7.26), septicemia (HR = 8.98; 95% CI: 6.84–11.79), gastrointestinal hemorrhage (HR = 3.60; 95% CI: 2.74–4.72), heart failure (HR = 4.88; 95% CI: 3.65–6.53), epilepsy or recurrent seizures (HR = 20.83; 95% CI: 12.24–35.43), and chronic kidney disease (HR = 2.73; 95% CI: 1.95–3.82) (all *p* < 0.05) (Table 4).

### 3.5. Time of the Occurrence of Postresuscitation Comorbidities

**Figure 2:**
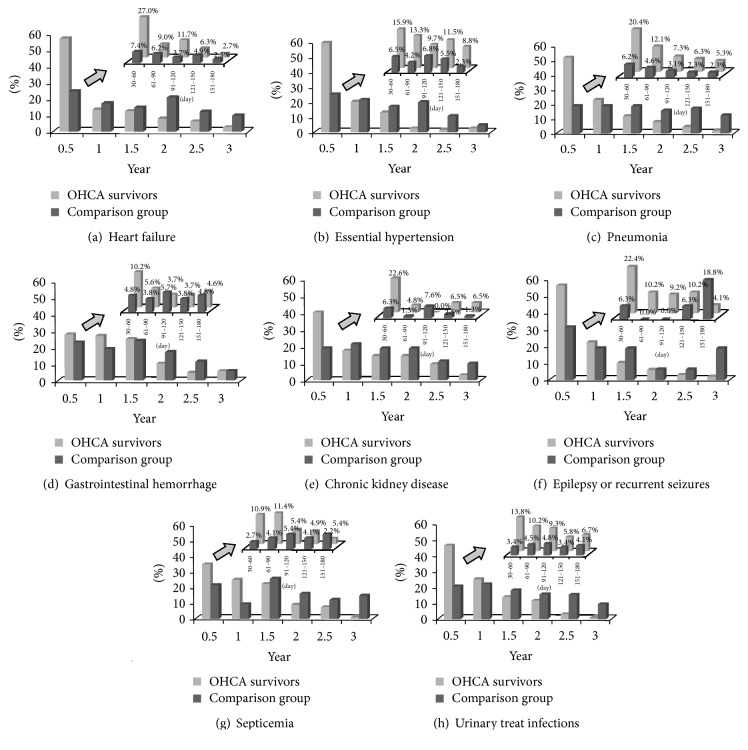
The prevalence of postresuscitation comorbidities in OHCA survivors. Figures (a) to (h) aim to show when these postresuscitation comorbidities develop during the study period; furthermore, each smaller figure aims to show the detailed prevalence in the first half year.

All OHCA survivors were followed to determine the timing of the development of new-onset postresuscitation comorbidities. Most comorbidities occurred in the first half year, including heart failure (56.8%), essential hypertension (59.3%), pneumonia (51.5%), chronic kidney disease (40.3%), epilepsy (56.1%), and urinary tract infections (45.8%).* In this study, we noted that 20.1% of pneumonia events occurred during the 30th day to 60th day after OHCA*. The prevalence of gastrointestinal bleeding was the highest (23.8%) during the period 1 to 1.5 years after OHCA (Figure 2). Finally, among the comparison patients, these diseases did not show a time-related distribution.

## 4. Discussion

Some previous studies have reported that the long-term survival rate of OHCA could be decreased by postresuscitation comorbidities [4, 27, 28]. However, the time points of the occurrence of comorbidities have never been well addressed. In this study, we followed long-term OHCA survivors (survival over one year) and found that most of the comorbidities occurred within the first half year after OHCA.

Among these postresuscitation comorbidities, we noted that cardiovascular diseases and infections were the most prevalent. The risk of suffering new-onset heart failure was clearly higher in OHCA survivors than in comparison patients (HR = 4.88; 95% CI: 3.65–6.53). Moreover, in the first half year, this prevalence among OHCA survivors was 32.1% higher than those in comparison patients. Several possible reasons could contribute to the high prevalence of heart failure. First, the procedure involved in providing chest compression (including manual or device-assisted compression) might not only force circulation but also induce secondary injury of the heart [29]. Some previous studies further demonstrated that myocardial damage, caused by direct physical force, was the second most common CPR-related injury [30–32]. This myocardial damage could influence cardiac contractility, which would increase the chances of heart failure [33–35]. Finally, the elevated levels of inflammatory mediators and nitric oxide production during the postresuscitation period might also cause myocardial injury and further induce heart failure [36, 37]. Therefore, early heart function surveys for preventing or treating heart failure in OHCA survivors might not be avoidable.

Infections were the most common comorbidities in OHCA survivors, in particular pneumonia developed in the early stages after OHCA. The first key factor associated with early pneumonia was brain hypoxic-ischemic injury, which presented early, and reasonably impaired swallowing function and gag reflex (resulting in aspiration pneumonia). In addition, rib fracture was the most common complication of chest compression [30]. These OHCA survivors might suffer from painful breathing and coughing, which would make it difficult to maintain respiratory tract hygiene. As a result, this could increase the chance of suffering from pneumonia [38, 39]. Some previous studies mentioned that early antibiotics for preventing pneumonia in OHCA patients might shorten the duration of hospital stay [40–42]. Unfortunately, prophylactic antibiotics for long-term treatment were not recommended because of a lack of strong evidence. Therefore, the duration of treatment with prophylactic antibiotics might need to be extended to cover this high-risk period (30th to 60th day after OHCA).

In addition to infections, we found that gastrointestinal hemorrhage was also a common postresuscitation comorbidity. Clinically, some treatments for critical cardiovascular events might potentially cause gastrointestinal hemorrhage (i.e., aspirin for acute coronary syndrome could cause peptic ulcer bleeding). Koskinas et al. also reported that gastrointestinal hemorrhage was both a major short-term and long-term complication in patients who received percutaneous coronary interventions [43]. Since peptic ulcers accounted for the majority of gastrointestinal hemorrhage cases, anticoagulation treatments (i.e., aspirin, heparin) for cardiovascular events should be treated more carefully [44]. Although epilepsy or recurrent seizures was not as common as infections or heart failure, the relative risk of these conditions was much higher in OHCA survivors than in comparison patients (HR = 20.83; 95% CI: 12.24–35.43). Almost a third of adult epilepsy cases are caused by brain damage, such as traumatic brain injury and stroke [45–47]. Therefore, we hypothesized that subsequent epilepsy in OHCA survivors could be related to hypoxic-ischemic brain damage during cardiac arrest. Finally, the rule regarding the provision of long-term antiepileptic agents to OHCA survivors was unclear. This study might support the routine treatment of antiepileptic agents in long-term postresuscitation care [48].

## 5. Limitations

One major limitation of this study is that the different functional levels of survivors were not be distinguished. Most of the comorbidities (including epilepsy, pneumonia, and urinary tract infections) that we identified to be more common in the study group might also be relatively common in patients admitted to rehabilitation facilities or long-term care wards. The conditions of survivors with good neurologic outcomes might differ obviously with those required long-term bed-ridden. Unfortunately, this database we used did not include the detailed neurologic outcome reports. In addition, because the same end point was established, the follow-up period for each OHCA survivor may not have been the same. Detailed quantitative or numeric laboratory reports were not included in this database. Therefore, we could not determine the severity of some complications. For example, this study was not able to identify the stage of chronic kidney disease, classification of heart failure, hemoglobin change in gastrointestinal bleeding, or blood pressure change in infectious disease. The OHCA survivors were not further classified to functional or nonfunctional survivors. The relationship between comorbidities and long-term neurologic outcomes is expected to be further analyzed in the future. Finally, because the facilities for OHCA management and long-term care clearly differ between different countries, the local database in Taiwan used in this study might not truly reflect the conditions in other countries.

## 6. Conclusions

Long-term OHCA survivors were at a high risk of suffering from heart failure, infections, gastrointestinal hemorrhage, and epilepsy in the postresuscitation period. Most importantly, most of the comorbidities occurred within the first half year after OHCA.

## Figures and Tables

**Table 1:** Characteristics and personal histories of OHCA survivors and comparison patients.

	OHCA survivors (*n* = 1,346)		Comparison patients (*n* = 4,038)		
	Number	%	Number	%	*p* value
Sex					1.000
Male	825	61.29	2475	61.29	
Female	521	38.71	1563	38.71	
Age (mean ± SD, y/o)	55.2 ± 22.9		54.3 ± 22.3		0.199
≦15	106	7.88	318	7.88	1.000
16–30	105	7.80	315	7.80	
31–45	172	12.78	516	12.78	
46–60	335	24.89	1005	24.89	
61–75	336	24.96	1008	24.96	
>75	292	21.69	876	21.69	
Personal history					
Cardiac diseases^*∗*^	202	15.01	195	4.83	<0.001
Diabetes^*∗*^	310	23.03	351	8.69	<0.001
Hypertension^*∗*^	423	31.43	718	17.78	<0.001
Renal failure^*∗*^	72	5.35	39	0.97	<0.001
Liver cirrhosis^*∗*^	16	1.19	11	0.27	<0.001
Stroke^*∗*^	52	3.86	56	1.39	<0.001
Economic level (monthly income in USD$)^*∗*^					<0.001
<600	415	30.83	1089	26.97	
601–1,000	680	50.52	2014	49.88	
>1,000	250	18.57	926	22.93	
Geographic region of Taiwan^*∗*^					
Northern	732	54.38	2018	49.98	<0.001
Central	281	20.88	890	22.04	
Southern	272	20.21	1013	25.09	
Eastern	60	4.46	108	2.67	
Urbanization					0.484
1 (most)	328	24.37	1032	25.56	
2	153	11.37	404	10.00	
3	368	27.34	1090	26.99	
4	496	36.85	1503	37.22	

OHCA: out-of-hospital cardiac arrest.

^*∗*^Significant differences.

**Table 2:** Postresuscitation comorbidities of OHCA survivors.

Comorbidities	OHCA survivors		Comparison patients		
	Number	%	Number	%	*p* value
Urinary tract infections^*∗*^	225	16.72	290	7.18	<0.001
Pneumonia^*∗*^	206	15.30	130	3.22	<0.001
Septicemia^*∗*^	184	13.67	74	1.83	<0.001
Essential hypertension	113	8.40	309	7.65	0.380
Heart failure^*∗*^	111	8.25	81	2.01	<0.001
Gastrointestinal hemorrhage^*∗*^	108	8.02	105	2.60	<0.001
Epilepsy or recurrent seizures^*∗*^	98	7.28	16	0.40	<0.001
Chronic kidney disease^*∗*^	62	4.61	79	1.96	<0.001

OHCA: out-of-hospital cardiac arrest.

^*∗*^Significant differences.

**Table 3:** Significant differences in the three most common postresuscitation comorbidities between OHCA survivors and comparison patients.

	Pneumonia						Septicemia						Urinary tract infections					
	OHCA survivors (*n* = 206)			Comparison patients (*n* = 130)			OHCA survivors (*n* = 184)			Comparison patients (*n* = 74)			OHCA survivors (*n* = 225)			Comparison patients (*n* = 290)		
	Number	%	*p* value	Number	%	*p* value	Number	%	*p* value	Number	%	*p* value	Number	%	*p*	Number	%	*p* value
Sex			0.103			0.361			0.871			0.395			0.940			0.001
Male	137	67		85	65		114	62		47	64		137	61		151	52	
Female	69	33		45	35		70	38		27	36		88	39		139	48	
Age group			0.146			0.000			0.036			0.000			0.088			0.000
≦60	99	48		32	25		83	45		12	16		105	47		108	37	
61–75	62	30		30	23		58	32		22	30		65	29		78	27	
>75	45	22		68	52		43	23		40	54		55	24		104	36	
Economic level (monthly income in USD$)			0.116			0.001			0.162			0.009			0.267			0.006
<600	76	37		44	34		67	36		28	38		78	35		98	34	
601–1,000	97	47		73	56		89	48		39	53		112	50		143	49	
>1,000	33	16		13	10		28	15		7	09		35	16		49	17	
Geographic region of Taiwan			0.305			0.815			0.387			0.085			0.065			0.037
Northern	101	49		64	49		94	51		28	38		111	49		129	44	
Central	50	24		26	20		36	20		17	23		48	21		82	28	
Southern	47	23		37	28		46	25		25	34		59	26		74	26	
Eastern	8	04		3	02		8	04		4	05		7	03		5	02	
Urbanization			0.237			0.047			0.315			0.002			0.501			0.025
1 (most)	60	29		22	17		55	30		12	16		61	27		61	21	
2	26	13		12	09		19	10		9	12		27	12		27	09	
3	54	26		34	26		46	25		11	15		53	24		70	24	
4	66	32		62	48		64	35		42	57		84	37		132	46	

**Table 4:** The risk of suffering from postresuscitation comorbidities in OHCA survivors.

Comorbidities	HR	95% CI	*p* value
Urinary tract infections^*∗*^	2.83	2.37–3.37	<0.001
Pneumonia^*∗*^	5.82	4.66–7.26	<0.001
Septicemia^*∗*^	8.98	6.84–11.79	<0.001
Essential hypertension	1.23	0.99–1.52	0.059
Heart failure^*∗*^	4.88	3.65–6.53	<0.001
Gastrointestinal hemorrhage^*∗*^	3.60	2.74–4.72	<0.001
Epilepsy or recurrent seizures^*∗*^	20.83	12.24–35.43	<0.001
Chronic kidney disease^*∗*^	2.73	1.95–3.82	<0.001

HR: hazard ratio; CI: confidence interval.

^*∗*^Significant differences between OHCA survivors and comparison patients.
